# AZIN1 RNA editing alters protein interactions, leading to nuclear translocation and worse outcomes in prostate cancer

**DOI:** 10.1038/s12276-022-00845-6

**Published:** 2022-10-06

**Authors:** Aram Ghalali, Liangzhe Wang, Konrad H. Stopsack, James M. Rice, Shulin Wu, Chin-Lee Wu, Bruce R. Zetter, Michael S. Rogers

**Affiliations:** 1 Vascular Biology Program and Department of Surgery, Boston Children’s Hospital, Harvard Medical School, Boston, MA USA; 2 Department of Medicine, Memorial Sloan Kettering Cancer Center, New York, NY USA; 3 Department of Pathology, Massachusetts General Hospital, Harvard Medical School, Boston, MA USA; 4 Present Address: Department of Pathology, Shanghai Changzheng Hospital, Shanghai, China; 5 Present Address: Silicon Therapeutics, Boston, MA USA

**Keywords:** Oncogenesis, Prostate cancer

## Abstract

The transcript encoding Antizyme Inhibitor 1 (AZIN1) is frequently edited in various cancers, and this editing is associated with enhanced tumor aggressiveness. After comparison of wild-type AZIN1 (wtAZIN1) and edited AZIN1 (edAZIN1, which contains a Ser367Gly substitution), we report differential binding of edAZIN1 to a small set of proteins; specifically, edAZIN1 binds to alpha-smooth muscle actin (ACTA2), gamma actin 1 (ACTG1), and myosin9, whereas wtAZIN1 does not. This binding enables nuclear translocation of edAZIN1. In contrast to overexpression of edAZIN1 and, to a lesser extent, (editable) wtAZIN1, overexpression of an uneditable AZIN1 allele does not promote a cellular phenotype associated with increased tumorigenicity. In patients, both editing and nuclear localization of AZIN1 are common and are associated with tumor aggressiveness, i.e., a higher Gleason score, higher genomic instability, and a shorter progression-free survival time. In conclusion, the data indicate that binding of edAZIN1 to the actin/myosin9 complex supports its nuclear translocation, leading to enhanced cellular aggressiveness, and is associated with worse prostate cancer outcomes.

## Prostate cancer: Tracing tumor aggressiveness to modified RNA

A hyperactive molecular ‘editing’ process that changes a single base in a messenger RNA molecule leads to a single amino acid change in the protein coded for by the RNA, initiating a series of interactions that can make prostate cancer more aggressive. The RNA codes for the protein Antizyme inhibitor 1 (AZIN1) and is frequently edited in ways that make a variety of cancers more aggressive. Work spearheaded by Aram Ghalali and led by Michael Rogers and Bruce Zetter at Harvard Medical School, Boston, USA, explored the consequences of AZIN1 RNA editing in prostate cancer. They found that the modified AZIN1 protein binds to three specific proteins, causing it to move into the cell nucleus. They suggest AZIN1 transport into the nucleus is a key event leading to poorer outcomes in patients with prostate cancer.

## Introduction

Many cancer types arise from changes in genetic information, among which somatic DNA mutations are the most recognized. In prostate cancer (PC), these DNA mutations include single nucleotide alterations, copy number variations^1^, and chromosomal rearrangements^2,3^. Recently, however, increasing evidence suggests that posttranscriptional modifications to RNA (composing the epitranscriptome) also have a crucial role in tumorigenesis^4^. RNA bases are modified in several different ways, among which RNA editing is the most common. The most prevalent type of human RNA editing, adenosine-to-inosine (A-to-I) conversion, is catalyzed by ADAR (adenosine deaminase acting on RNA), with ADAR1 amplification and upregulation being associated with increased tumor aggressiveness and poor patient outcomes^5^ in multiple human cancers.

An ADAR-catalyzed A-to-I conversion in a single codon of AZIN1 mRNA causes a serine-to-glycine substitution at residue 367, resulting in edited AZIN1 (edAZIN1). A pan-cancer analysis revealed that a small number of nonsynonymous A-to-I RNA edits may constitute a key driver event and have a critical functional role in different tumor contexts. In this analysis, the event associated with aggressive tumor subtypes, more advanced stages, and worse survival profiles in the greatest number of cancers was AZIN1 editing. Furthermore, the expression of edAZIN1 increases resistance to several chemotherapeutic agents^6^. In hepatocellular carcinomas, this editing event is associated with cytoplasmic-to-nuclear translocation of AZIN1, and a relatively low level of AZIN1 editing was shown to be sufficient to confer an aggressive phenotype^7^. Similar findings were shown in esophageal squamous cell carcinoma^8^, as well as colon^9,10^, gastric^11^ and lung carcinomas^12^. However, the mechanism by which a single amino acid substitution in the AZIN1 protein, resulting from a single base substitution in its mRNA, leads to such a substantial gain-of-function phenotype and increased tumorigenesis remains uncertain.

Several potential mechanisms underlying the altered functionality of edAZIN1 have been hypothesized, but few data exist to support any of these hypotheses^7–12^. These hypotheses include the following: 1. altered functionality of edAZIN1 as a result of its translocation to the nucleus; 2. altered (increased) binding affinity of edAZIN1 for its principle known binding partner, antizyme; and 3. altered interactions with yet-unidentified proteins that bind to edAZIN1 but not to wild-type AZIN1. Clearly, an improved understanding of the phenotypic differences between edited and wild-type AZIN1 will provide important insights into ways to abolish its oncogenic function.

In this report, we investigate the consequences of AZIN1 RNA editing in prostate cancer. We show that editing of AZIN1 mRNA leads to altered binding to a select group of proteins and that these interactions influence the ability of the edited form of the protein to enter the nucleus. Blocking these interactions prevented nuclear accumulation of AZIN1 as well as the acquisition of cellular phenotypes associated with increased tumor aggressiveness.

Furthermore, the presence of edAZIN1 in the nucleus in human prostate cancer samples correlates with markers of tumor progression and with poor oncologic outcomes. This work expands the known role of AZIN1 editing in human cancer and further suggests novel approaches to interfere with edAZIN1-mediated tumor aggressiveness.

## Materials and methods

### Cell culture

Cell lines were purchased from ATCC (Manassas, VA). PC3, DU145 (both prostate carcinoma) and HEK293 (human embryonic kidney) cells were grown in RPMI-1640 medium (Gibco, 11875, NY, USA), DMEM, and DMEM (Gibco, 11885), respectively; both media were supplemented with 10% FBS, penicillin‒streptomycin (Gibco 15140), and 1 mM L-glutamine (A2916801).

wtHEK293T and ADAR1 KO HEK293T cells^13^ were a generous gift from Dr. Charles M. Rice at Rockefeller University, NY, USA. These cells were grown in DMEM supplemented with 10% FBS, 1X nonessential amino acids and penicillin‒streptomycin.

### Confocal microscopy

Cells were fixed with 4% formaldehyde and analyzed with an LSM 880 META confocal laser scanning microscope (Zeiss, Oberkochen, Germany) equipped with 63x and 40x A-Plan oil immersion objectives using ZEN imaging software in multitrack mode.

### Generation of the uneditable AZIN1 construct

The codon corresponding to amino acid residue 367 in AZIN1 was mutated from AGC (Ser) to TCC (Ser). The mutations were introduced by site-directed mutagenesis using a QuikChange Lightning Site-Directed Mutagenesis Kit according to the manufacturer’s protocol (Agilent Technologies). The forward primer GATCAAATTGTGGAATCCTGTCTTCTTCCTGAGCTGAATGTGGG and the reverse primer CCCACATTCAGCTCAGGAAGAAGACAGGATTCCACAATTTGATC were used.

### Protein expression and purification

The bacterial expression vectors Clover-pBAD (Addgene, #54575), mRuby2-pBAD (Addgene, #54771), and pBAD-mTAG-BFP2 (Addgene, #54572) encoding the 6X-HIS-tagged fluorescent protein with a TEV protease cleavage site were purchased from Addgene. The human OAZ1 gene was codon optimized for expression in *E. coli* (https://www.idtdna.com/CodonOpt) and synthesized de novo (ThermoFisher). The DNA sequences encoding the N-terminal fluorescent fusion protein of hAZIN1 and the *E. coli* expression-optimized hOAZI were synthesized by overlap extension PCR using previously described methods^14^. Expression vectors of C-terminal fluorescent fusion proteins were synthesized using NEBuilder HiFi DNA Assembly (New England Biolabs). Point mutations in the hAZIN1 gene were introduced using PCR-based site-directed mutagenesis with Pfu Ultra High-Fidelity DNA Polymerase (Agilent) using the described protocols. The fusion proteins were expressed and purified according to our previously described methods^15^.

To generate mammalian expression vectors, we amplified the full-length cDNA sequences encoding Clover-Antizyme and Clover-edited-antizyme from the pBAD expression vectors described above by PCR with the primers

F: 5’- cgcGCTAGCcGccATGgTGAAATCCTCCCTGCAGcg-3’ and

R: 5’-cgcAAGCTTCTTACTTGTACAGCTCGTCCATCC-3’, and used the primers

F: 5’- cgcGCTAGCcGccATGgTGAGCAAGGGCGAG-3’ and

R: 5’-cgcAAGCTTTTATGCTTCAGCGGAAAAGCTGTC-3’ to similarly amplify the Antizyme-mRuby2 sequence. Subsequently, the purified products were ligated into pcDNA3.1 + (Addgene, MA, USA) according to the manufacturer’s instructions. The other plasmids used, pcDNA3.1-Clover-mRuby2 (plasmid #49089) and pcDNA3 mRuby2 LIC cloning vector (6H), were commercially available (Addgene, MA, USA).

All sequences were verified by multiple Sanger sequencing runs using forward and reverse primers (Eton Biosciences) and analyzed with 4peaks software. Gene, primer, and vector sequences can be found in the supplementary information (Supplementary Table 3).

### FRET Assay

The AZIN1-antizyme binding affinity was measured by a FRET assay as previously described^16^. In brief, “purified recombinant protein stocks in 50% glycerol were diluted with TBS (pH 7.4) + 0.15% Tween-20 to 2X final working concentration (100 nM for Clover containing donor proteins and 2 µM mRuby2 acceptor proteins) and transferred to a 96-well plate (Corning 3821). Specifically, acceptor protein or diluent control was transferred into a low-volume 384 well plate (CoStar 3356) in triplicate and serially diluted. Plates were equilibrated at room temp for 1 h before reading.

Fluorescence was measured using an EnVision plate reader (Perkin Elmer) with a 470/40 excitation filter and either a 515/30 or 600/8 emission filter to measure Clover or mRuby-2 fluorescence, respectively. Curve fitting on donor fluorescence (quenching) was performed in GraphPad Prism v. 7, using global fitting for maximum and minimum fluorescence intensity, constraining the Hill coefficient to 1, and fitting the IC_50_. Similar IC_50_s were obtained when fitting was performed on acceptor sensitization after excluding acceptor concentrations above 100 nM. In our in vitro FRET determination for AZIN and antizyme, we observed 17% donor quenching and the quantum yield for mRuby2 is 0.38, predicting a 6.4% acceptor sensitization”.

### Droplet Digital PCR (ddPCR)

The ddPCR primers Forward (GAGCCTCTGTTTACAAGCAG) and Reverse (CATGGAAAGAATCTGCTCCC) and probes wtAZIN (5’-/5HEX/GCTCAGGAAGAAGACAGCTTTCCAC/3IABkFQ/-‘3) and edAZIN (5’-/56-FAM/GCTCAGGAAGAAGACAGCCTTCCA/3IABkFQ/-‘3) used in this study were designed using Primer3 software to target AZIN. Droplet digital PCR was performed as follows. The PCR mixture contained ddPCR Super Mix (Bio-Rad, Hercules, CA; final concentration: 1X), the wtAZIN and edAZIN probes (0.25 μM each), forward and reverse primers (1 μM each), and up to 30 ng of template DNA in a 25 μL total volume. The reaction mixture was emulsified into approximately 16–17,000 droplets using a QX100 Droplet Generator (Bio-Rad) according to the manufacturer’s instructions. PCR was performed (10 min. at 98 °C; 40 cycles of 30 s at 94 °C, 60 s at 58 °C, and 20 s at 72 °C; 10 min at 72 °C; and holding at 12 °C); the samples were analyzed within 24 h using a QX100 Droplet Reader (Bio-Rad), and the data were analyzed with QuantaSoft software (Bio-Rad).

### RNA extraction and Illumina mRNA library preparation

HEK293 cells were transfected for 24 h with plasmids expressing fluorescent protein (Empty); wild-type AZIN1 (wt), which can be edited endogenously; pseudoedited AZIN1 (ed); or AZIN1 with an uneditable codon 367 (uneditable-wt). RNA was extracted using a Qiagen kit and was then sequenced and analyzed by Macrogen as described^17^.

### Western Blotting

Protein concentrations in lysates were quantified, and proteins were separated by sodium dodecyl sulfate–polyacrylamide gel electrophoresis (SDS‒PAGE). After separation, proteins were transferred onto a polyvinylidene difluoride (PVDF) membrane (Bio-Rad Laboratories, Inc. and incubated with a primary antibody followed by a secondary antibody. Luminescence was visualized with Enhanced Chemiluminescence Substrate (ECL) (Amersham Biosciences, Sweden). The results of western blotting were analyzed with NIH ImageJ 1.62 software. The following antibodies were used: anti-ACTG1 (actin gamma 1) and anti-ACTA2 (alpha-smooth muscle actin) from Sigma–Aldrich, anti-AZIN1 from Abbexa, and anti-ADAR1, anti-Myosin-9, anti-GAPDH, anti-α-Tubulin, anti-snail, anti-slug, anti-MMP2, anti-MMP9 and anti-Rpb1 CTD from Cell Signaling.

### Mysoin-9 CRISPR knockout cells

We digested lentiCRISPRv2 blast (Addgene, cat# 98293) and/or lentiCRISPRv2 hygro (Addgene, cat# 98291) with the BsmB1 restriction enzyme (NEB cat# R0739S) at 55 °C for 1 h and performed gel purification (0.7 g agarose in 100 ml of TAE buffer) using a gel extraction kit (NEB cat# T1020S). sgRNAs were selected using the ChopChop website (https://chopchop.cbu.uib.no/). Oligos/sgRNAs were purchased from Eton Bioscience (Supplementary Table 4) and were phosphorylated by the addition of T4 PNK in T4 DNA ligase buffer (37 °C for 50 min). Thereafter, the oligos were annealed by holding at 95 °C for 5 min followed by ramping down to 25 °C at 5 degrees/minute. The oligos and plasmids were ligated by incubation with Quick Ligase for 10 min at RT and were then transformed into DH5alpha (NEB cat# C2987I) following the manufacturer’s instructions. Following sequence confirmation and purification with a maxiprep kit (Qiagen), the plasmids together with envelope pVSV-G plasmid (Addgene #138479) and packaging psPAX plasmid (Addgene #12260) were transfected into HEK295T cells for 24 h, and the medium containing lentivirus was then changed and extracted after an additional 24 and 48 h, respectively. DU145 and PC3 cells were exposed to the lentivirus-containing medium for 4 h, and fresh medium was then added to the cells for an additional 24 h. Antibiotic selection was performed by treating the cells with 5 ng/µl blasticidin and/or 250 µg/µl hygromycin for 4 days. Myosin 9 knockout was confirmed by western blotting.

### ADAR1 CRISPR knockout cells

The ADAR1 genomic locus was modified, which resulted in abolishing ADAR1 expression in the HEK293T cell line, by using CRISPR-Cas9 genome editing as described earlier^13^.

### Soft agar colony formation assays

A total of 5 × 10^3^ cells in 0.4% Bacto agar were seeded on top of a solidified layer of 0.6% Bacto agar in 6-well plates. Colonies consisting of more than 50 cells were counted after 19 days, and the data are expressed as the mean ± s.e.m. of triplicate wells in the same experiment.

### Matrigel invasion assay

We performed the invasion assay using 24-well BioCoat Matrigel Invasion Chambers (BD Biosciences) according to the manufacturer’s instructions. Briefly, 2 × 10^5^ cells were seeded in the top compartment, and DMEM containing 10% FBS was added to the bottom compartment as a chemoattractant. After 24 h of incubation, cells that invaded the Matrigel were fixed and stained with crystal violet (Sigma‒Aldrich). Cells were counted in 10 fields of view under a 20× objective and imaged using SPOT imaging software (Nikon, Japan).

### Coimmunoprecipitation (Co-IP)

HEK293 cells were transfected with 2.0 μg of the Clover-tagged wild-type or edited AZIN1 plasmid (GFP-wtAZIN1 or GFP-edAZIN1) and analyzed via Co-IP using a V5-specific antibody. Approximately 10 mg of the total cell lysate was immunoprecipitated with 5 μg of an anti-FLAG antibody at 4 °C overnight. Immunocomplexes were then precipitated using 100 μl of protein G-agarose, which was provided in the immunoprecipitation kit (Roche Diagnostics Co., Indianapolis, IN). After extensive washing in washing buffer, the beads were boiled in 50 μl of loading buffer and analyzed by western blotting using antibodies against myosin-9 (cell signaling), ACTG1 (actin gamma 1-), and ACTA2 (alpha-smooth muscle actin) from Sigma–Aldrich. We utilized 5% of the total lysate (5% input) as a positive control. Mouse immunoglobulin G (Santa Cruz Biotechnology) was used as a negative control.

### Patients and tissue microarray (TMA)

Prostate cancer patients (*n* = 202) who underwent radical prostatectomy at Massachusetts General Hospital (Boston, MA) between September 1993 and March 1995 were included. Patients who received neoadjuvant hormonal treatment or adjuvant hormonal and/or radiation treatment before recurrence were excluded. The Gleason score was reassigned based on the current International Society of Urological Pathology (ISUP) recommendation^18^. TMAs were constructed as previously described^19^. Index tumor foci from each case were selected for inclusion in the TMA along with 26 adjacent benign tissues. The study protocol was approved by the human study committees at MGH (IRB# 2005P000774). For the high Gleason score cohort, patients who underwent radical prostatectomy for localized PC between 1993 and 2007 were reviewed, and patients with Gleason scores of 7 or higher were included in this study.

### Immunohistochemical analysis

TMAs were deparaffinized with xylene, rehydrated, and subjected to brief proteolytic digestion and peroxidase blocking. Slides were incubated overnight at 4 °C with a 1:400 dilution of a polyclonal anti-antizyme inhibitor 1 antibody (#11548–1-AP; Proteintech Group, Inc. Chicago, IL). The antibody was validated using cells overexpressing AZIN1 plasmids. After washing, a peroxidase-labeled polymer and the substrate chromogen TMB were used to visualize the staining of the proteins of interest (DAKO EnVision System, Dako Diagnostics, Zug, Switzerland). Normal prostate tissues were used as controls. Slides were scored independently by two board-certified pathologists blinded to the patient data, and discrepancies were resolved through concurrent re-examination of the slide to arrive at a consensus. The percentage of cells showing nuclear or cytoplasmic AZIN staining was tabulated; specimens were scored as positive if >3% tumor cells exhibited immunoreactivity.

### Statistical analyses

In the tissue microarray cohort, PSA failure-free survival was defined as the time between the initial surgery and the appearance of detectable PSA in patients with two consecutive increases in PSA, and metastasis-free survival was defined as the time to clinical or radiographic detection of metastasis.

Data from The Cancer Genome Atlas (TCGA) Prostate Adenocarcinoma (PRAD) cohort of patients with primary prostate cancer^20^ were obtained using previously described methods^21^. Analyses were restricted to the set of *n* = 333 tumors with high-quality RNA sequencing data. The edAZIN1 level was quantified by Han et al.^6^ in *n* = 292 tumors. All tumors with missing edAZIN1 calls had missing PSA values but otherwise similar characteristics to tumors with edAZIN1 calls. Grading of cribriform morphology was performed as described in^22^.

To quantify associations between edAZIN1 and mRNA expression levels, Pearson correlation analysis was used after log-transforming mRNA levels. Predictors of high edAZIN1 levels (ADAR mRNA expression, fraction of the genome altered, age at diagnosis, and AZIN1 mRNA level) were assessed using multivariable linear regression, and all predictors were standardized to interquartile ranges (25-percentile increase) for comparability between predictors.

For survival analyses in the tissue microarray cohort and the TCGA cohort, Cox proportional hazards regression analysis was used to estimate hazard ratios and 95% CIs. Multiplicative effect measure modification was evaluated using the Wald test for the product term (*p*_interaction_).

## Results

### Overexpression of edAZIN1 results in an aggressive cancer cell phenotype

**Fig. 1:**
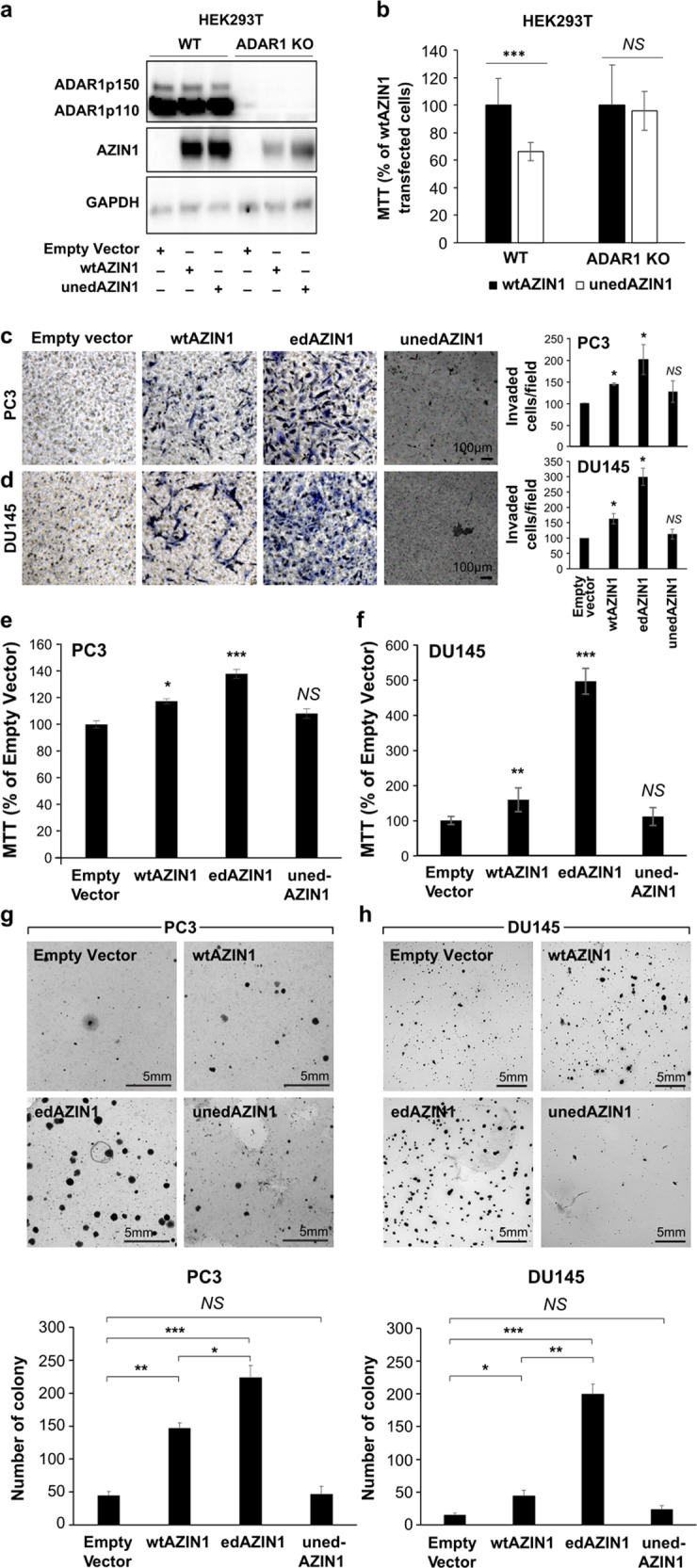
edAZIN1 increases prostate cancer cell aggressiveness. The importance of the unedAZIN1 control was established by overexpressing wtAZIN1 or unedAZIN1 (2.5 µg plasmid for 24 h) in wtHEK293T and ADAR1 KO HEK293T cells. The samples were analyzed (**a**) by western blotting with the indicated antibodies and (**b**) with an MTT assay. The effect of overexpressing various AZIN1 alleles on the behavior of human prostate cancer PC3 or DU145 cells with regard to (**c** and **d**) invasion into the extracellular matrix, **e** and **f** cell proliferation, and (**g** and **h**) colony formation in soft agar. **a**–**h** PC3 and DU145 cells (as indicated in the figure) were transfected with plasmids expressing pCDNA3.1 (Empty); wild-type AZIN1 (wtAZIN1), which can be edited endogenously; pseudoedited AZIN1 (edAZIN); or AZIN1 with an uneditable codon 367 (uneditable AZIN1). Proliferation was measured by MTT reduction. Invasion through Matrigel supported by a Transwell membrane (8 μm pores) was assessed after methanol fixation and toluene blue staining. Soft agar colony formation was assessed by incubation for 19 days, fixation, and crystal violet staining. In (**b**–**h**), the bars indicate the mean (±S.D.) from three independent experiments. * significant difference compared to empty vector (*P* < 0.05), (or compared to wtAZIN1 as in (**b**)), ** significant difference compared to empty vector (*P* < 0.01), *** significant difference compared to empty vector (*P* < 0.001).

To determine whether editing of AZIN1 mRNA results in protein “gain of function”, we transiently overexpressed three different forms of AZIN1 in prostate cancer cells. In wtAZIN1, the AGC (Ser) codon at residue 367 can be deaminated by ADAR to IGC (Gly). Although exogenous expression of wild-type (wt) AZIN1 is frequently used as a control for comparison with edAZIN1, wtAZIN1 RNA may be edited by endogenous ADAR1 in tumor cells. To provide an improved control, we generated an uneditable AZIN1 (unedAZIN1) gene by changing codon 367 from AGC to TCC, which encodes serine but does not contain an adenosine ribonucleotide base. This control was added, as we observed that cells transfected with wtAZIN1 had an increase in edited AZIN1 mRNA, indicating that a fraction of wtAZIN1 was targeted by ADAR1. Additionally, in ADAR1 KO HEK293T cells, overexpression of either wtAZIN1 or unedAZIN1 had similar effects on cell proliferation, while compared with the unedAZIN1 allele, the wtAZIN allele significantly increased the cellular proliferation of wtHEK293T cells (Fig. 1a, b), confirming that wAZIN1 might be edited by endogenous ADAR1. PC3 cells were transfected with an expression vector encoding the wtAZIN1, uneditable-wtAZIN1 (TCC codon), or edAZIN1 (GGC codon) protein or with the empty vector control, and changes in global transcription were determined by RNAseq. Individually altered genes are presented in supplementary file 1. Principal component analysis (PCA) revealed that compared to overexpression of the pre-edited or wild-type AZIN1 vector (which can be edited), transfection with the uneditable-wtAZIN1 vector led to negligible differences with respect to the empty vector control (Supplementary Fig. 1), suggesting that edAZIN1 induces transcriptional changes distinct from those induced by unedited AZIN1. We next sought to determine whether the presence of edAZIN1 causes increased tumorigenic potential using the PC3 and DU145 prostate cancer cell lines. We found that only constructs capable of coding for edited AZIN1 increased cancer cell aggressiveness, as determined by increases in proliferation, invasiveness, and anchorage-independent colony growth (Fig. 1c−h). These data suggest that only the edited form of AZIN increases the tumorigenic potential and that even a low frequency of AZIN1 editing (as occurs with wtAZIN1)^7^ may be sufficient to promote tumorigenic potential if sufficient ADAR1 is present.

### Editing of AZIN1 is sufficient to drive its nuclear localization

**Fig. 2:**
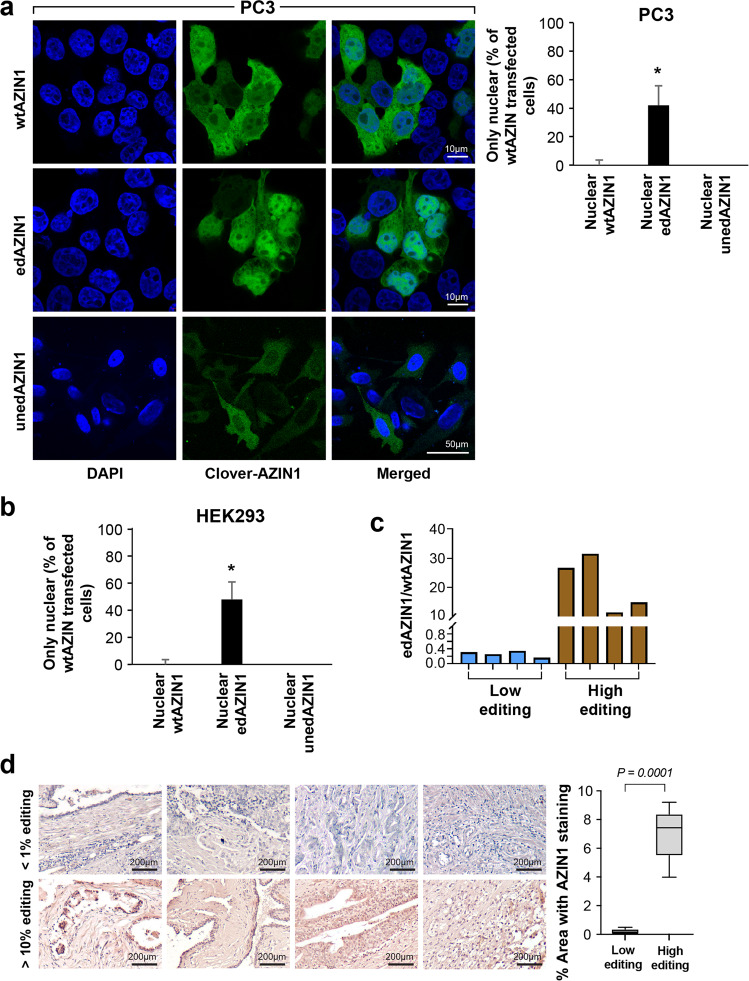
RNA editing of AZIN1 induces its nuclear localization. **a**, PC3 and (**b**) human embryonic kidney (HEK293) cells were transfected with 1.5–2.5 µg of pcDNA3.1-Clover-AZIN1, pcDNA3.1-Clover-edited-AZIN1 or Clover-uneditable-AZIN1 for 24 h and analyzed by confocal microscopy. **c** The amount of edAZIN1 was measured by ddPCR, and tissues with low and high AZIN1 editing were identified (tissues with low editing had < 1% edAZIN1 among total AZIN1 RNA, while tissues with high editing had up to 31.5%). Then, the same tissues were stained with an anti-AZIN1 antibody (**d**). Tissues with low AZIN-1 editing showed cytoplasmic localization only (up), while tissues with high AZIN1 editing showed cytoplasmic and nuclear localization of AZIN1 (down). The intensity was measured by ImageJ software, and the anti-AZIN1 antibody was validated as described in the Materials and Methods section. In (**a** and **b**), the bar graphs summarize the percentages of 50 cells exhibiting intense nuclear localization of antizyme + /− 95% confidence intervals. Measurements were performed in a blinded manner. In each case, nuclear localization was significantly associated with transfection with the edAZIN plasmid (*P* < 0.0001 by Fisher’s exact test).

We further investigated whether the amino acid substitution in edAZIN1 could drive the nuclear localization of AZIN1 in prostate cancer^7^. Separate vectors encoding wtAZIN1, uneditable-wtAZIN1, or edAZIN1 were constructed to express a functional fusion protein with an N-terminal fluorescent Clover tag to allow visualization^16^ and transfected into PC3 and HEK293 cells. In both cell lines, the wtAZIN1 fusion protein was observed either in the cytoplasm or distributed ubiquitously throughout the cell, consistent with the prominent cytoplasmic immunostaining of endogenous AZIN1 in these cell lines (Supplementary Fig. 2a, b). In contrast, exogenous expression of edAZIN1 resulted in strong nuclear localization (Fig. 2a, b), and the fusion protein was observed only in the nucleus in almost half of the transfected cells. This finding demonstrates that the single amino acid substitution found in edAZIN1 (S367G) is sufficient to localize AZIN1 to the nucleus.

We next investigated the cellular localization of AZIN1 in sections from human prostate cancer tumors by IHC staining following confirmation of the presence of edAZIN1 mRNA by droplet digital PCR (ddPCR) (Fig. 2c). We found that AZIN1 localized to cell nuclei within tissues with higher frequencies of AZIN1 editing (Fig. 2d). Taken together, our data indicate that the single S367G substitution in AZIN1 caused by RNA editing is sufficient to induce nuclear localization of AZIN1 and support the conclusion that the nuclear AZIN1 observed in the prostate cancer sections was the edited form (Fig. 2c).

### Overexpressed edAZIN1 and wtAZIN1 interact with different proteins

**Fig. 3:**
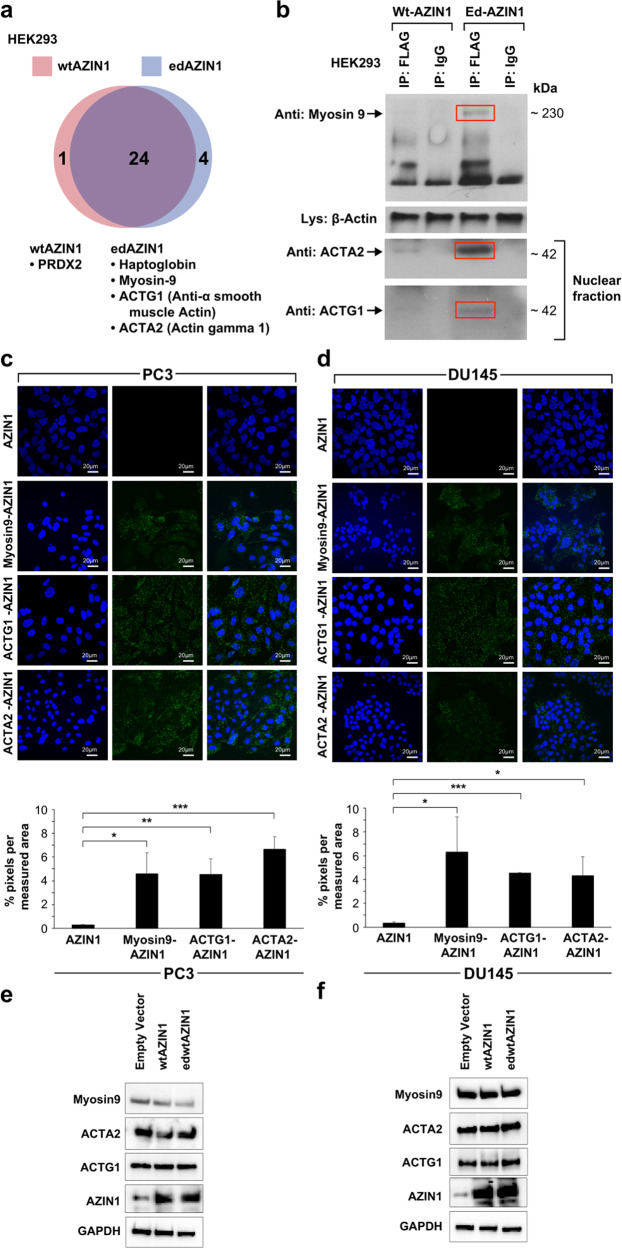
Differential interactomes of wtAZIN1 and edAZIN1. **a** Scheme by which candidate differential interactors were identified. HEK293 cells were transfected with FLAG-wtAZIN1 or FLAG-edAZIN1 and immunoprecipitated with an anti-FLAG antibody. We separated the immunoprecipitated complexes by gel electrophoresis and stained the gel with Coomassie blue. The bands were analyzed by mass spectrometry, and 268 proteins were identified. After removal of nonspecific binding (IP not greater than IgG (*p* < 0.05 counts, or >10-fold difference in intensity sum), we found 5 proteins that selectively interact with either edAZIN1 or wtAZIN1. **b** Western blotting was performed to confirm differential interactions. HEK293 cells were transfected with FLAG-wtAZIN1 or FLAG-edAZIN1 (24 h), immunoprecipitated with an anti-FLAG antibody or IgG and analyzed for myosin9 expression by western blotting. Other confirmed edAZIN1-selective interactors included ACTG1 and ACTA2 in the nuclear fractions. **c** and **d** Proximity ligation assay for detecting binding between Myosin-9, ACTG1 or ACTA2 and AZIN1 in the indicated cell lines. The anti-AZIN1 antibody alone (first panel) was used as a negative control. wtAZIN1 and edAZIN1 were overexpressed in PC3 (**e**) and DU145 (**f**) cells, and samples were analyzed for Myosin-9, ACTG1 and ACTA2 expression by western blotting.

**Fig. 4:**
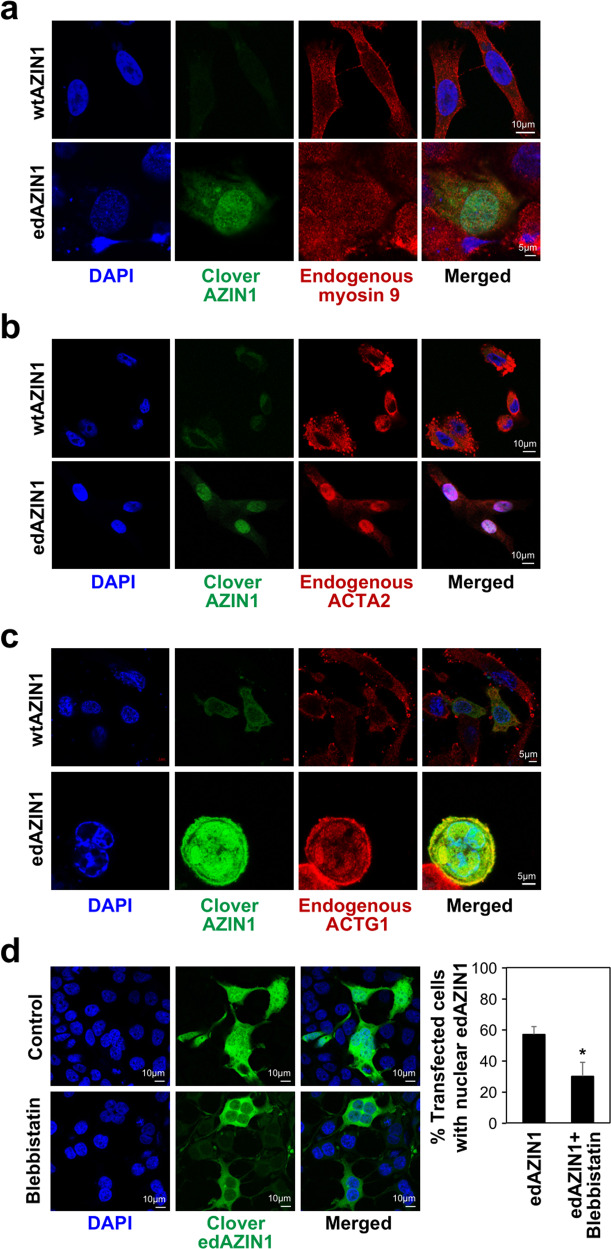
The myosin 9-actin complex translocates edAZIN1 into the nucleus. Confocal micrographs of PC3 cells transfected with Clover-wtAZIN1 or Clover-edAZIN1 (24 h) and stained with anti-mysosin-9 (**a**), anti-ACTA2 (**b**) or anti-ACTG1 (**c**) antibodies with DAPI counterstaining. **d** Confocal micrographs of HEK293 cells transfected with Clover-edAZIN1 (24 h) and treated with 50 µM blebbistatin for the entire transfection time with DAPI counterstaining. The bars indicate the mean (±S.D.) from three independent experiments. * significant difference compared to edAZIN1-transfected cells (*P* < 0.05).

To develop a mechanistic understanding of the oncogenic properties of edAZIN1, we hypothesized that unedited AZIN1 and edAZIN1 have different binding partners. To test this hypothesis, HEK293 cells were transfected with N-terminal FLAG-tagged wtAZIN1 or edAZIN1 and immunoprecipitated with an anti-FLAG antibody. The immunoprecipitated fraction bound to FLAG was analyzed by liquid chromatography–tandem mass spectrometry (LC‒MS/MS), and peptides from 4 proteins were identified specifically under the edAZIN1-expressing condition (Fig. 3a); in addition, 3 of these interactions were further confirmed by coimmunoprecipitation followed by western blotting (Fig. 3b). The specific edAZIN1-interacting proteins included myosin-9, alpha-smooth muscle actin (ACTA2) and gamma actin 1 (ACTG1). This result is of particular interest because the myosin-9-actin protein complex was previously shown to have a role in the nuclear translocation of other proteins^23,24^. Using proximity ligation assay, we confirmed that each of these proteins associates with AZIN1 in PC3 (Fig. 3c) and DU145 (Fig. 3d) prostate cancer cells. The protein levels of myosin9, ACTG1 and ACTA2 were not altered after overexpression of the AZIN1 alleles (Fig. 3e, f). Immunofluorescence staining of these proteins in PC3 cells (Fig. 4a–c) revealed that myosin9, ACTG1 and ACTA2 were localized predominantly in the cytoplasm in both nontransfected and wtAZIN1-overexpressing cells. In cells overexpressing edAZIN1, however, myosin-9 was localized to both the cytoplasm and nucleus (Fig. 4a). Importantly, both ACTG1 and ACTA2 colocalized with edAZIN1 in the nucleus (Fig. 4b, c). These results demonstrate that edAZIN1 but not wtAZIN1 is capable of forming a complex with myosin9 and colocalizing with alpha-actin 2 and gamma actin 1.

### Myosin-9 is required for nuclear localization of edAZIN1 and the invasive phenotype

**Fig. 5:**
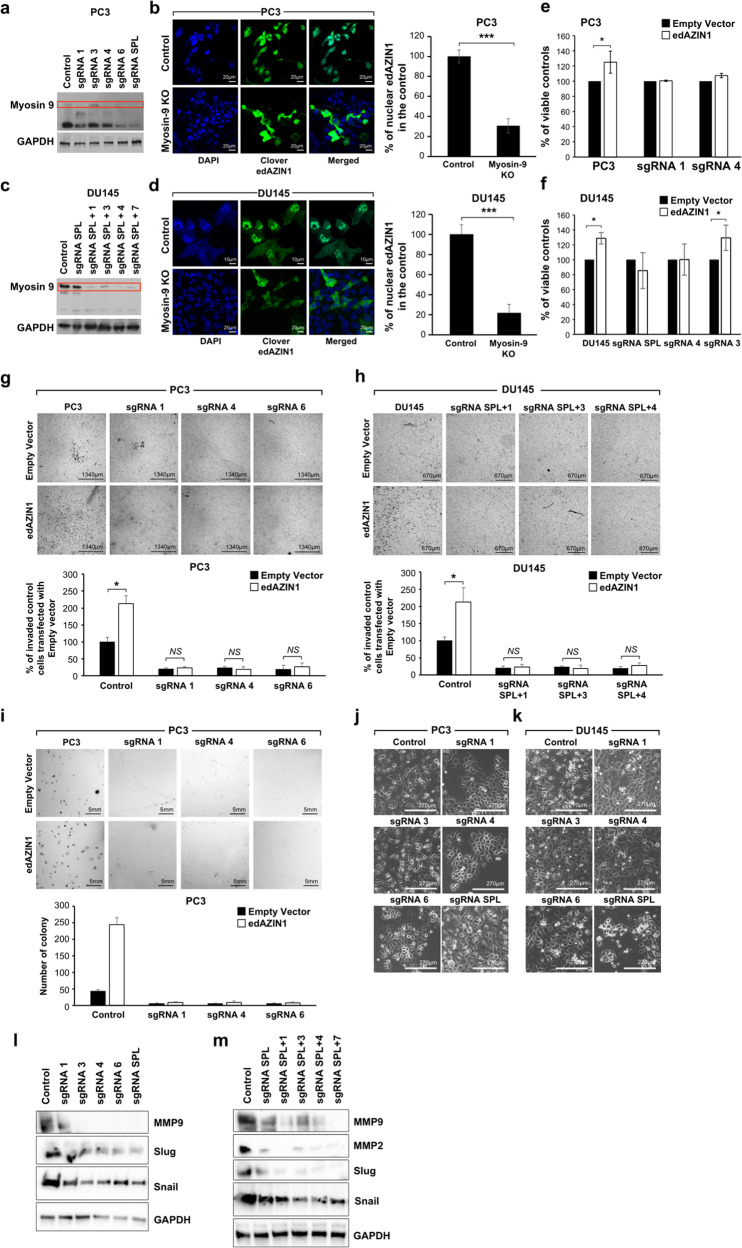
edAZIN1 fails to induce an aggressive phenotype in Myosin9 knockout cells. Using CRISPR/Cas9 gene editing and specific sgRNAs, Myosin 9 protein expression was successfully knocked out in PC3 (**a**) and DU145 (**c**) cells. Myosin 9-knockout PC3 (**b**) and DU145 (**d**) cells were transfected with 2,5 µg of pcDNA3.1-Clover-edited-AZIN1 for 24 h and analyzed by confocal microscopy. The effect of overexpressing the edAZIN1 allele in Myosin 9 knockout PC3 and DU145 cells with regard to cell proliferation (**e** and **f**), invasion into the extracellular matrix (**g** and **h**), and colony formation in soft agar (**i**). Proliferation was measured by MTT reduction. Invasion through Matrigel supported by a Transwell membrane (8 μm pores) was assessed after methanol fixation and toluene blue staining. Soft agar colony formation was assessed by incubation for 19–21 days, fixation, and crystal violet staining. The morphology of PC3 (**j**) and DU14 (**k**) cells is shown, and representative images acquired by light microscopy (lens: original magnification, 10X) are presented. The bars indicate the mean (±S.D.) from three independent experiments. * significant difference compared to empty vector (*P* < 0.05), ** significant difference compared to empty vector (*P* < 0.01) and *** significant difference compared to empty vector (*P* < 0.001). Myosin 9 knockout PC3 (**l**) and DU145 (**m**) cell populations were analyzed by western blotting with the indicated antibodies.

To investigate the role of myosins in the nuclear localization of edAZIN1, PC3 cells were treated with blebbistatin, a potent pan-myosin inhibitor^25^. This reduced the nuclear localization of edAZIN1 (Fig. 4d) by 40–50%. Next, we used CRISPR to specifically evaluate the effect of myosin9. We knocked out the myosin-9 gene in PC3 and DU145 prostate cancer cells. As shown in Fig. 5a, c, myosin-9 was successfully targeted using several different guide RNAs in both cell lines. Importantly, when edAZIN1 was overexpressed in myosin-9 KO PC3 and DU145 cells, it remained localized to the cytoplasm, in contrast with the nuclear localization observed in the isogenic controls (Fig. 5b, d). These results suggest that the nuclear localization of edAZIN1, a hallmark of its expression in cancer cells and a correlate of tumor aggressiveness, is mediated by myosin9. Furthermore, edAZIN1 overexpression increased cell proliferation (Fig. 5e, f), invasion (Fig. 5g, h) and colony-forming capacity (Fig. 5i), but this effect was not observed in Myosin-9 KO cells, further supporting the requirement of Myosin-9 for the influence of edAZIN1 on the cellular phenotype. Interestingly, we also observed that successful myosin-9 ablation distinctly altered cell morphology, with the spindle-shaped mesenchymal-like morphology becoming more epithelial in appearance (Fig. 5j, k). This morphological change was in line with the considerable decreases in the levels of the epithelial-to-mesenchymal transition markers snail and slug and the invasiveness markers MMP2 and MMP9 (Fig. 5l, m).

### S367G mutation does not increase the binding affinity of AZIN1 to antizyme

**Fig. 6:**
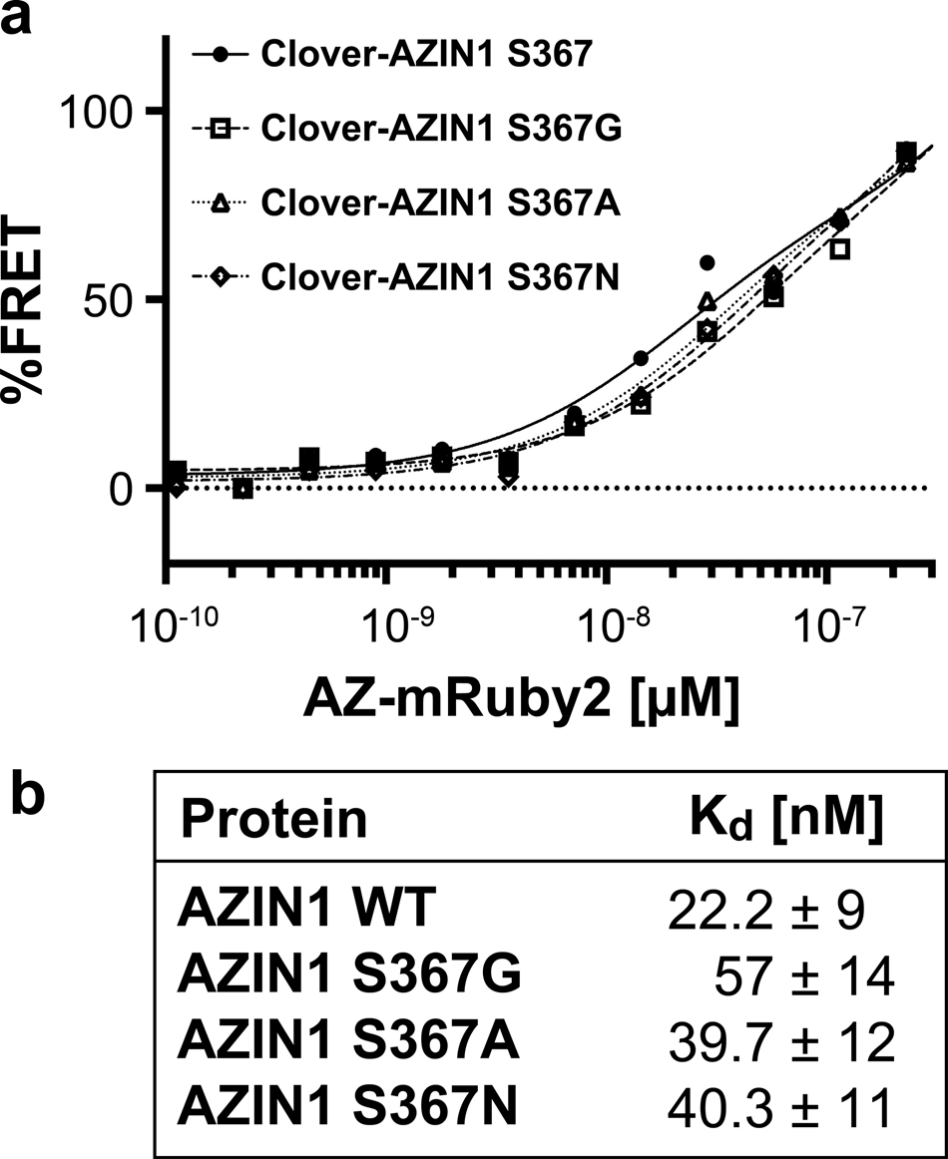
Mutational analysis was performed to measure the contribution of residue 367 in AZIN1 to the binding affinity for antizyme (AZ). **a** The binding affinities of AZ for 3 AZIN1 position 367 point mutants were measured by a FRET assay with titration of AZ-mRuby2 (100 pM-1 µM) to a fixed concentration of Clover-AZIN1 variants (50 nM). **b** The data were fitted using nonlinear regression to a four-parameter binding isotherm to determine the equilibrium dissociation constant (Kd) of the protein‒protein interaction.

In previously reported co-IP experiments, an increased interaction between edAZIN1 and antizyme was observed. This result has been interpreted as indicating an increased binding affinity of edAZIN1 for antizyme relative to that of wtAZIN1^7^. To measure the dissociation constant (K_d_) directly, we purified recombinant fusion proteins and used the Förster resonance energy transfer (FRET) ratio as a measure of the binding interaction^16^. The S367G substitution modestly weakened the binding affinity for antizyme to 57 nM compared with 22 nM for the wild-type protein (Fig. 6a, b), suggesting that the increased interaction of edAZIN1 with antizyme observed in co-IP experiments is not due to a higher binding affinity of the edited form of the protein.

To further understand the AZIN1-antizyme structure-affinity relationship, we tested the hypothesis that substituting the hydroxymethyl side chain of serine 367 with a smaller hydrogen at position 367 might allow greater conformational flexibility in the “switch 1” region of the AZIN1 protein, allowing it to bind more tightly to the antizyme protein^26^. We substituted serine 367 with either alanine, which contains a nonpolar methyl group, or asparagine, which contains a larger 2-carbon carboxamide side chain, to allow greater or less conformational flexibility at position 367, respectively. We observed similar K_d_ values of 39 nM and 40 nM for the protein‒protein binding interaction between antizyme and the S367A mutant (39 mM) and S367N mutant (40 mM) AZIN1 (Fig. 6a, b). This result indicates that although position 367 may be in a switch region and important for the binding of AZIN1 to antizyme, a mutation at this position does not alter the affinity of AZIN for the antizyme protein and suggests that this mechanism does not account for the tumorigenic effect of edAZIN1.

### Nuclear expression and RNA editing of AZIN1 is associated with worse prognosis in prostate cancer

**Fig. 7:**
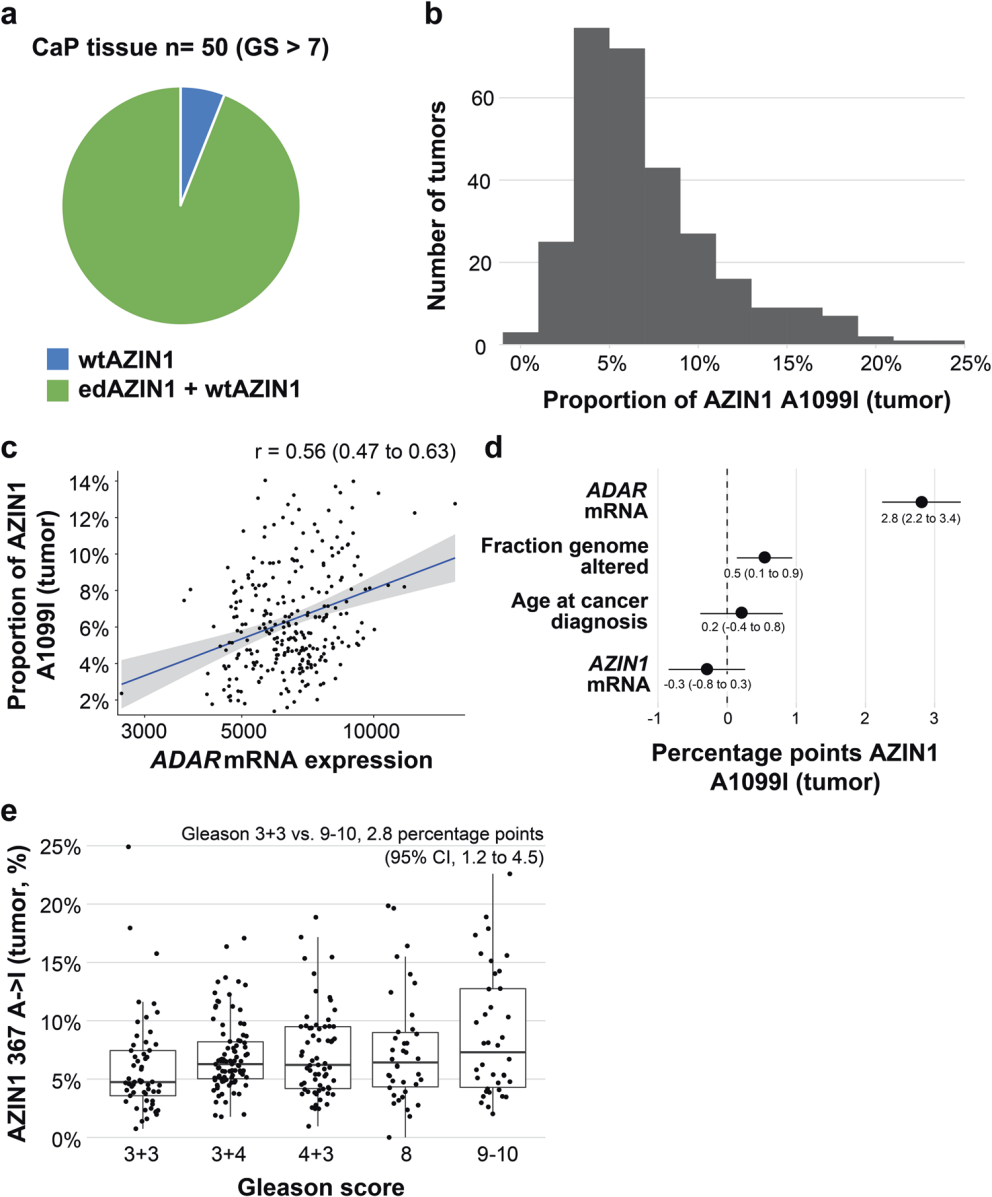
AZIN1 editing is common in prostate cancer. **a** Prostate cancer tissues (Gleason score > 7) were analyzed for wtAZIN and edAZIN mRNA levels by ddPCR. **b** Analysis of data from primary prostate cancer patients available via The Cancer Genome Atlas (TCGA) for the distribution of AZIN1 editing levels (*n* = 333). **c** Correlation between the relative RNA levels of ADAR1 and edited AZIN1 in primary prostate cancer patients from the TCGA cohort (*n* = 291 with edAZIN1 calls). Frequency of AZIN1 editing in prostate cancer patients stratified by age at cancer diagnosis and genomic instability level (**d**) and Gleason score (**e**) (TCGA cohort, *n* = 333).

To establish the clinical relevance of AZIN1 editing in prostate cancer, we studied different patient populations using three orthogonal methods. First, we investigated the prevalence of edAZIN1 in 50 human prostate cancer samples with a Gleason score > 7 using droplet digital PCR (ddPCR). The edited AZIN1 mRNA transcript was detected in 94% of the 50 prostate cancer samples tested (Fig. 7a). Although we previously showed that knockdown of AZIN1 decreases prostate tumor growth in vivo^27^, this is the first time that enhanced expression and a high frequency of AZIN1 RNA editing has been shown in human prostate cancer tissues.

Second, we analyzed the association between edAZIN1 and progression-free survival in The Cancer Genome Atlas prostate cancer cohort (Supplementary Table 1). We restricted the analyses to primary prostate tumors with high-quality RNA sequencing data, of which 292 were assessed for edAZIN1. A median of 6.1% of AZIN1 sequencing reads were A- > I edited (edAZIN1; interquartile range, 4.4 to 8.9) (Fig. 7b). In the multivariate logistic models, the strongest predictor of a higher edAZIN1 level was higher ADAR1 expression (by 2.8% per interquartile range increase in ADAR1 expression; 95% CI, 2.3 to 3.4) (Fig. 7c). edAZIN1 levels were also higher in tumors with higher genomic instability (Fig. 7d) and in tumors with a higher Gleason score (Fig. 7e). Specifically, we observed a 0.5 percentage point increase in the edAZIN1 level per interquartile range increase in copy number alteration burden (95% CI, 0.1 to 0.9) and a 2.8 percentage point difference in the edAZIN1 level between Gleason score 3 + 3 and 9–10 tumors (95% CI, 1.2 to 4.5). Together, our results strongly suggest that edAZIN1 is common in primary prostate cancer and is associated with higher ADAR1 expression and features of aggressive tumors.

**Fig. 8:**
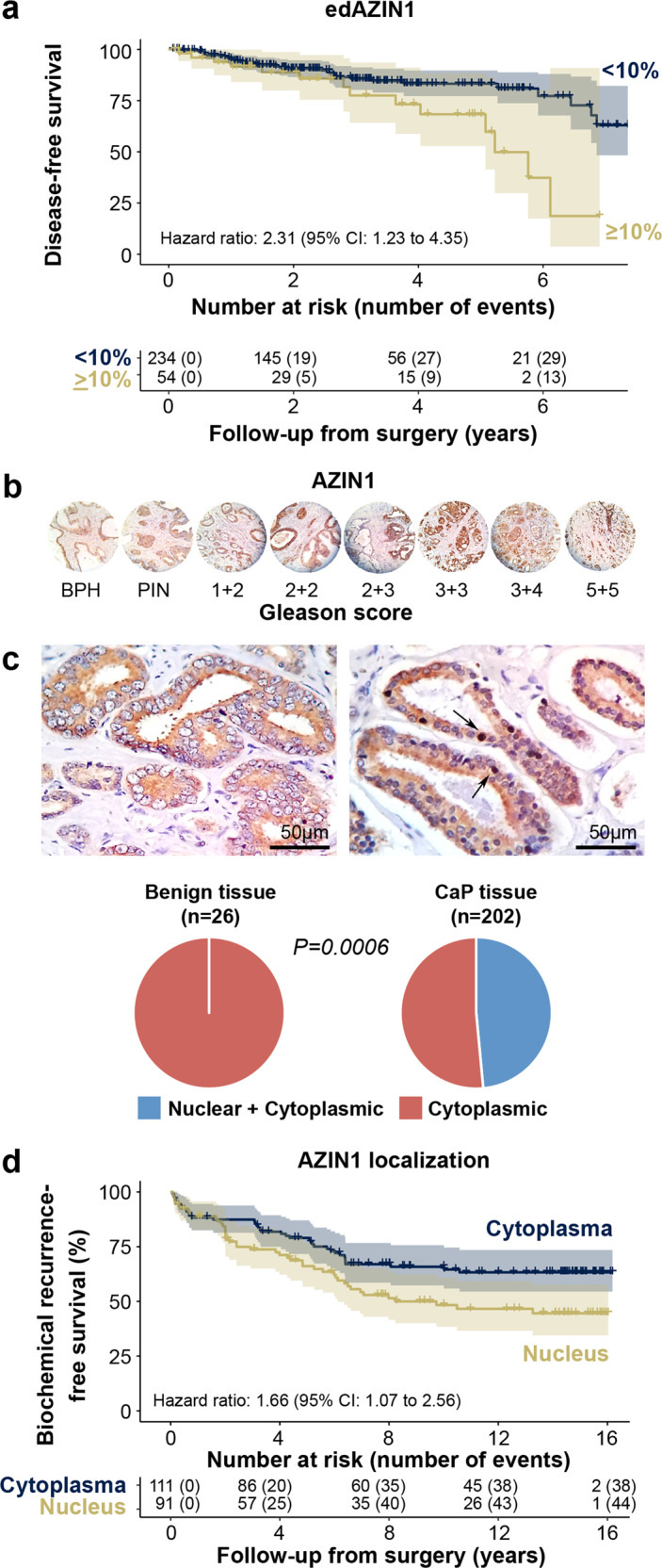
AZIN1 expression and nuclear localization in prostate cancer tissue. **a** Association between edAZIN and progression-free survival in The Cancer Genome Atlas prostate cancer cohort (*n* = 292). In (**b** and **c**) Representative IHC images of prostate tissue microarrays stained with a validated anti-AZIN1 antibody. **b** TMA images arranged by increasing Gleason score. **c** Benign Prostate tissue showing cytoplasmic localization only (left) or Prostate Cancer tissue showing both cytoplasmic and nuclear localization of AZIN1 (right, arrows indicate nuclear localization). The recurrence-free survival rate is plotted for (**d**) patients diagnosed with prostate cancer (*n* = 202). Patents were followed -up for up to 16 years.

Next, a total of 288 prostate cancer patients were followed over a median of 32 months, during which time 46 experienced disease progression (mostly biochemical recurrence; Fig. 8a). A high edAZIN1 level (≥10% edited *AZIN1* mRNA identified by RNA-sequencing) was associated with a shorter progression-free survival time (unadjusted hazard ratio, 2.31; 95% CI, 1.23 to 4.35) than was a low edAZIN1 level (<10%) (Fig. 8a). Adjusting for Gleason score (adjusted hazard ratio, 1.92; 95% CI, 1.00 to 3.68) or for age at diagnosis and fraction of the genome altered did not substantially attenuate this association (adjusted hazard ratio, 1.83; 95% CI, 0.95 to 3.5). Cribriform morphology is a histologic pattern that is increasingly recognized as a reflection of specific molecular features in prostate cancer^22^. Interestingly, in an exploratory, non-hypothesis-driven analysis, the association between high edAZIN1 mRNA levels and progression-free survival (PFS) was more pronounced in tumors without cribriform morphology (hazard ratio, 8.4; 95% CI, 1.98–36) than in tumors displaying cribriform morphology (hazard ratio, 1.83; 95% CI, 0.83–4.0; *p*_interaction_ = 0.06). Taken together, these results demonstrate an association between AZIN1 RNA editing and an increased incidence of tumor progression in prostate cancer patients.

Finally, we analyzed tissue microarrays of radical prostatectomy samples from 202 patients with prostate cancer and 26 adjacent benign prostate tissue samples by IHC staining (Fig. 8b, c). High AZIN1 expression was observed in both the benign samples (10/26; 38%) and tumor samples (91/202; 45%), suggesting that overall AZIN1 protein expression may not be a suitable biomarker to differentiate between tumor and benign tissue (Supplementary Table 2). In contrast to AZIN1 expression, the subcellular AZIN1 localization differed markedly between the tumor and benign control samples, with 98/202 tumor samples (49%) and 0/26 benign samples (0%; *p* < 0.001) exhibiting nuclear AZIN1 localization. Furthermore, nuclear localization was associated with a higher Gleason score (Fig. 8b). Taken together, these findings strongly suggest that the subcellular localization of AZIN1 may be a more robust marker for prostate cancer than total AZIN1 expression (Fig. 8c).

**Fig. 9:**
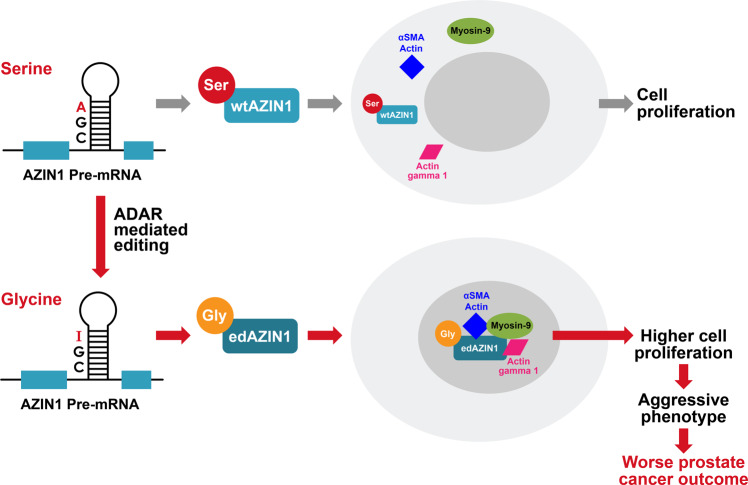
The role of edAZIN1 in prostate cancer. The oncoprotein AZIN1 stimulates cell proliferation (wild type AZIN1) (gray arrow). However, ADAR-mediated adenosine-to-inosine (A → I (in red)) editing of AZIN1 transcripts results in a serine (Ser) to glycine (Gly) substitution at residue 367. The RNA edited AZIN1 binds to Myosin 9, Actin gamma 1 and Anti-alpha smooth muscle Actin. Editing also causes nuclear localization of AZIN1, leads to more aggressive cell behavior (increased proliferation, invasion, and soft agar colony formation), and predicts worse prostate cancer outcome.

This patient cohort was additionally monitored over a 16-year follow-up period post-surgery (median, 14.1 years), during which time 82 men experienced biochemical recurrence of their prostate cancer. Compared to patients with tumors not exhibiting nuclear AZIN1 expression, tumors from patients showing nuclear AZIN1 were more likely to experience relapse after surgery (hazard ratio, 1.66; 95% CI, 1.07–2.56; Fig. 8d). Nuclear AZIN1 expression was not associated with either the presence or absence of cribriform morphology. In an unbiased analysis, the association between nuclear AZIN1 expression and biochemical recurrence was stronger among tumors without cribriform morphology (hazard ratio, 2.28; 95% CI, 1.36–3.81) than among those with cribriform morphology (hazard ratio, 0.63; 95% CI, 0.25–1.57; *p*_interaction_ = 0.013). No such difference was observed when stratifying by low vs. high Gleason score. While the number of metastatic events was low (26 events over a median of 15.0 years of follow-up), the overall association between nuclear AZIN1 expression and metastasis-free survival was of similar direction and magnitude as that for biochemical recurrence (hazard ratio, 1.98; 95% CI, 0.91–4.31). In summary, among multiple cohorts, AZIN1 editing and nuclear localization were associated with a high Gleason score, genetic instability, increased metastasis, and poor outcomes, especially in tumors not displaying cribriform morphology. It is likely that these poor outcomes are caused by changes in the interaction of AZIN1 with myosin9 and/or actins, together with the nuclear localization that results from editing (Fig. 9).

## Discussion

Deamination of a single adenosine base in AZIN1 mRNA and the resultant single amino acid change have been independently confirmed to have a remarkable phenotypic impact in multiple cancer types. As first shown in hepatocellular carcinoma^7^, ADAR1-mediated AZIN1-mRNA editing is associated with increased tumor aggressiveness in a variety of human cancers and is positively correlated with proliferation, migration and invasion in multiple cell lines^8–12,28^. Indeed, among all editing sites assessed, A-to-I conversion in the single codon corresponding to residue 367 in AZIN1 is associated with clinical features of aggressive cancer subtypes and is particularly interesting^6^. However, the role of AZIN1 in human prostate cancer has not been explored in depth. We found that the presence of edAZIN1 induces increased proliferation, invasion, and colony formation of prostate cancer cells, whereas the presence of an uneditable AZIN1 allele did not. These results highlight the essential role of ADAR1-mediated editing in AZIN1-induced aggressiveness and provide additional context for the importance of ADAR1 as an oncogene. Importantly, this conclusion could not be drawn without the use of the uneditable control, because wild-type cDNAs are susceptible to editing, and even low levels of editing may be sufficient to increase the tumorigenic potential, as shown, for example, in HCC cells^7^.

On the basis of coimmunoprecipitation of overexpressed proteins, it was previously suggested that edAZIN1 binds with higher affinity to antizyme than does wtAZIN1^7^. However, this technique does not measure direct interactions or affinity. To directly measure the affinity between AZIN1 variants and antizyme, we developed a FRET assay^7,16^. Surprisingly, we found that editing did not increase the affinity of AZIN1 for antizyme, suggesting that the functional differences in edAZIN1 are unlikely to be due to changes in its affinity for antizyme. In contrast, we found that edAZIN1 does bind preferentially over wild-type AZIN1 to a small number of proteins, specifically myosin-9, ACTG1, and ACTA2. This result is intriguing because the actin-myosin-9 complex has previously been reported to function as a nuclear shuttling complex, translocating multiple proteins to the nucleus to target the transcription machinery^23,24^. Interference with myosin-9 in an in vivo environment has also been shown to regulate a number of cancer hallmarks, including invasion, growth and EMT^29–31^. However, the role of edAZIN1 has not yet been investigated in those models. Blocking an essential component of the shuttling complex, myosin9, through pharmacologic inhibition resulted in the retention of edAZIN1 in the cytoplasm. We confirmed this by disrupting the myosin-9 gene in PC3 and DU145 cells using CRISPR‒Cas9 gene editing. As hypothesized, edAZIN1 was excluded from the nucleus in myosin-9 knockout cells.

Controlling for both the expression of the AZIN1 isoform and the myosin-mediated shuttling of edAZIN1 also allowed us to explore how nuclear localization of AZIN1 influences cellular behaviors that are known to predict tumor aggressiveness. We found that myosin-9 CRISPR-knockout cells, where overexpressed edAZIN1 was restricted to the cytoplasm, did not exhibit an increase in proliferation, invasion, or anchorage-independent colony formation upon edAZIN1 overexpression, further suggesting that the localization and not the cellular expression level of edAZIN1 is responsible for the aggressive cellular phenotype. This is the first time that both the presence of AZIN1 and its nuclear localization have been associated with increased tumor aggressiveness and clarifies that this association is due to high RNA editing. Our work directly supports the hypothesis that only edAZIN1 is translocated to the nucleus and that this translocation is necessary for the protumorigenic phenotype seen in edAZIN1-expressing tumor cells. At this time, the mechanism by which nuclear edAZIN1 promotes tumor aggressiveness remains unknown and is an important topic for future investigation.

Finally, for the first time, we quantified the extent to which edAZIN1 may contribute to tumor aggressiveness in patients with prostate cancer. We observed a striking difference in AZIN1 localization in clinical specimens of primary prostate cancer compared to benign paired control samples and observed a correlation between the editing and nuclear localization of AZIN1. This confirms the in vitro observation that editing of AZIN1 is required for its nuclear localization. Furthermore, we found that AZIN1 editing is common in primary prostate tumors, confirmed the increased presence of edAZIN1 in high Gleason score (7+) prostate cancers, and observed that increased editing of AZIN1 is associated with a higher Gleason score at the time of resection. We also showed here that edAZIN1 is correlated with higher genomic instability, increased metastasis, and poorer outcomes. Together, these data indicate that AZIN1 editing is a common mechanism by which prostate cancers develop enhanced aggressiveness. Importantly, during longitudinal follow-up in two independent cohorts of men with primary prostate cancer, increased AZIN1 editing and nuclear localization were associated with a higher risk of tumor recurrence following surgery. Taken together, these observations emphasize that nuclear localization of edAZIN1 is a feature of clinically aggressive prostate cancer.

In summary, the work presented here provides several previously unknown findings. These are: 1. RNA-edited AZIN1 binds to certain proteins that wild-type AZIN1 does not; 2. binding of edAZIN1 to an actin-myosin complex is associated with the translocation of these proteins to the nucleus; 3. nuclear translocation of edAZIN1 is required for increased tumor aggressiveness; and 4. AZIN1 is upregulated in human prostate cancers, and this upregulation correlates with increased tumor aggressiveness (Fig. 9). The present work strongly supports further exploration of nuclear edAZIN1 as a predictive biomarker for disease outcomes in patients with prostate cancer. Additionally, it highlights the importance of RNA editing in general and AZIN1 editing in particular as an underexplored mechanism linking RNA editing to aggressive phenotypes in human cancer. Together, our findings suggest that the S367G amino acid substitution in edAZIN1 leads to an interaction with myosin-9 and subsequent nuclear translocation, which is required for the acquisition of an altered, aggressive cellular phenotype. The role of edAZIN1 in the nucleus remains to be determined. However, this new insight into the requirement for edAZIN1 to enter the nucleus to exert its effect provides a rationale for targeting AZIN1 expression, editing or localization as a potential therapeutic approach in multiple cancer types.

## Supplementary information

Supplemental material

Supplemental file 1
