# Supplementary material for: AZIN1 RNA editing alters protein interactions, leading to nuclear translocation and worse outcomes in prostate cancer
Source: Exp Mol Med. 2022 Oct 6;54(10):1713–26. doi: 10.1038/s12276-022-00845-6 (PMC9636422; doi:10.1038/s12276-022-00845-6)
Supplement: Supplementary file 1 — Supplemental material [file 12276_2022_845_MOESM1_ESM.pdf]

Supplementary Fig. 1.

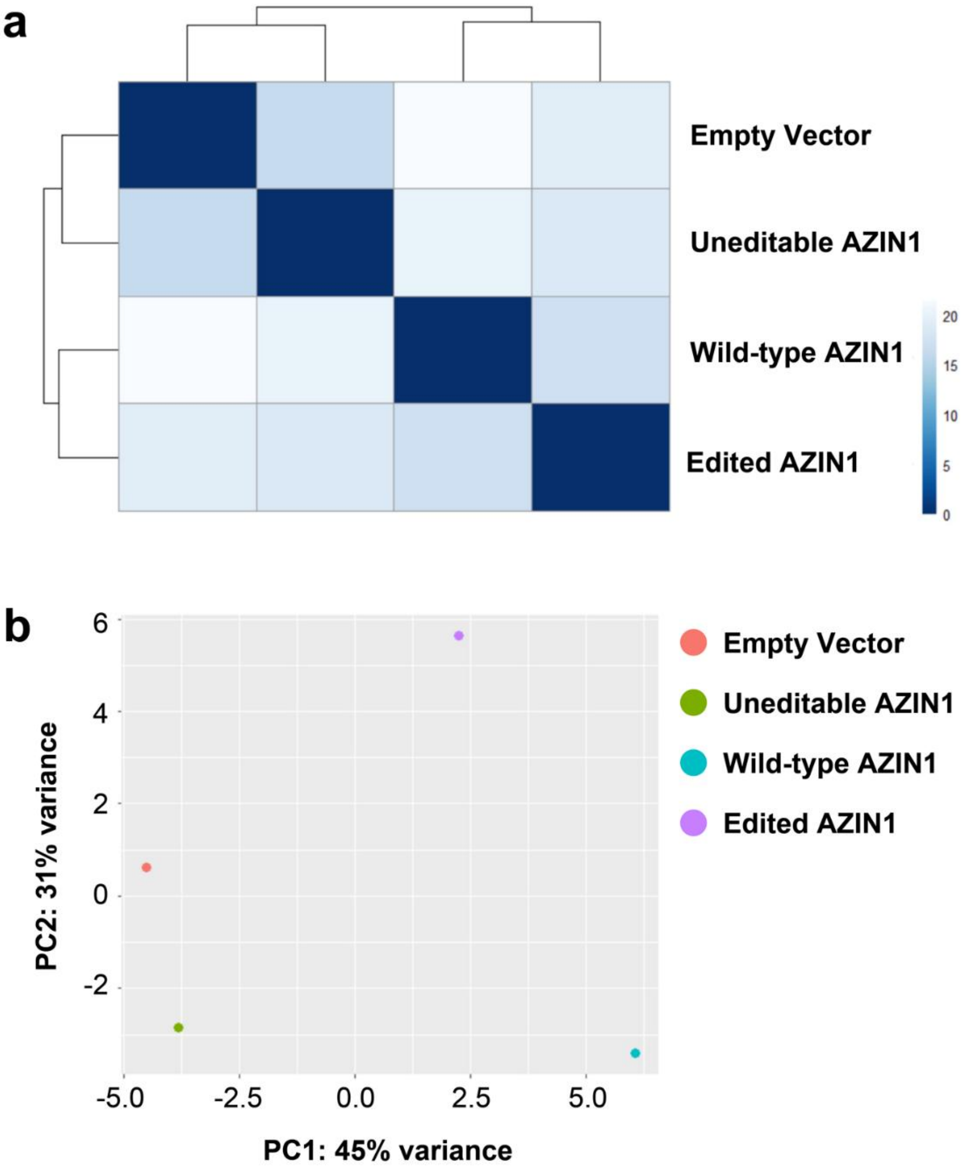

Supplementary Fig. 2.

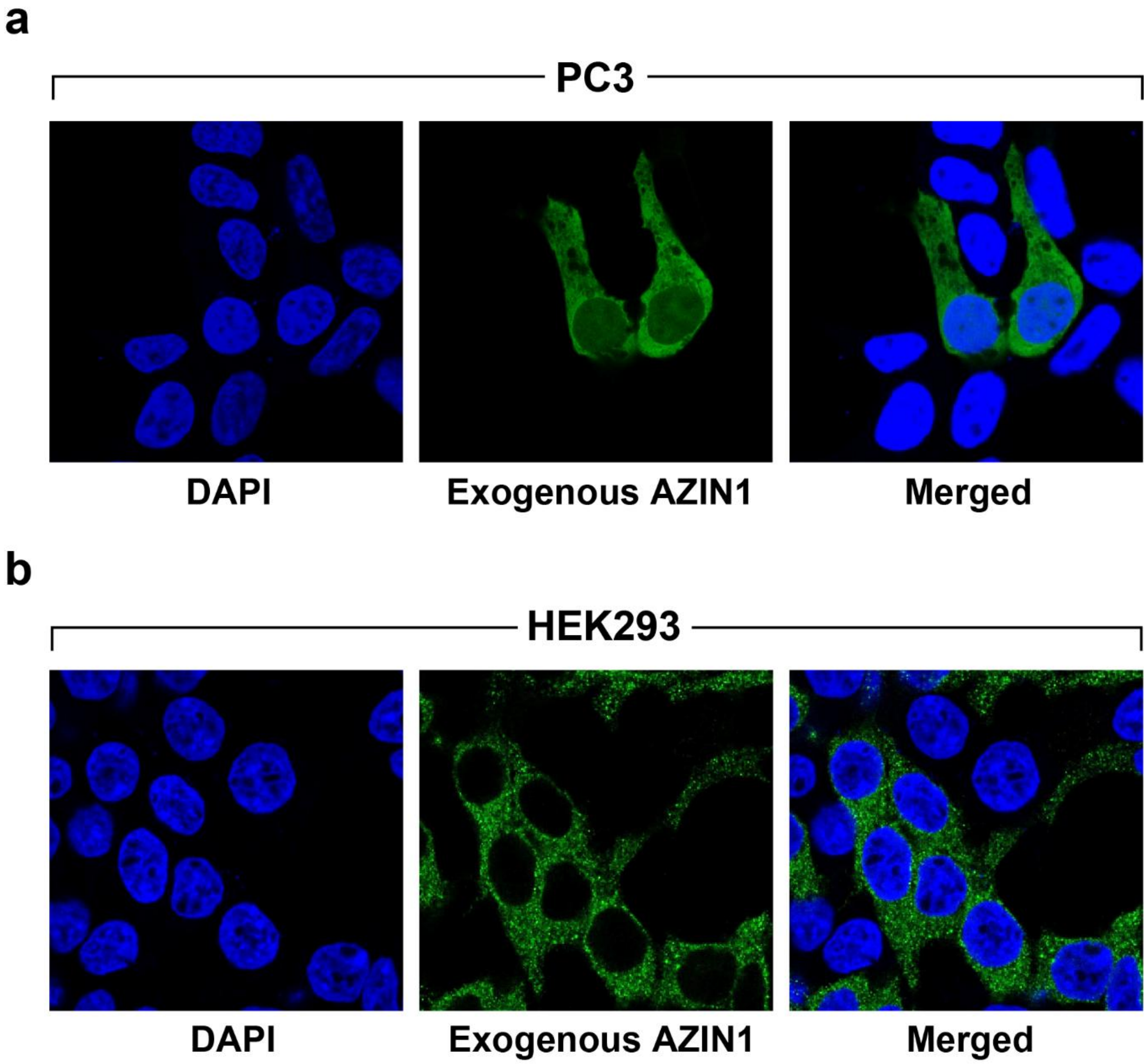

Supplementary Table 1.

Characteristic	Overall	By AZIN1 367 A->I (tumor)			
		<5%	5-<10%	10-<15%	15-25%
**N**	292	105	132	35	20
**Age at cancer diagnosis (years)**	61 (56, 66)	60 (56, 65)	61 (55, 66)	63 (57, 67)	64 (58, 66)
**Self-reported race**					
Asian	6 (2%)	2 (2%)	1 (1%)	2 (6%)	1 (6%)
African descent	39 (14%)	15 (15%)	19 (15%)	4 (12%)	1 (6%)
White	238 (84%)	86 (83%)	110 (85%)	27 (82%)	15 (88%)
Unknown	9	2	2	2	3
**PSA at diagnosis [ng/dl]**	7 (5, 12)	6 (4, 12)	7 (6, 11)	8 (5, 12)	15 (5, 26)
Unknown	105	36	50	12	7
**Gleason score**					
3+3	57 (20%)	33 (31%)	17 (13%)	4 (11%)	3 (15%)
3+4	94 (32%)	23 (22%)	56 (42%)	13 (37%)	2 (10%)
4+3	65 (22%)	23 (22%)	31 (23%)	7 (20%)	4 (20%)
8	38 (13%)	14 (13%)	16 (12%)	4 (11%)	4 (20%)
9-10	38 (13%)	12 (11%)	12 (9%)	7 (20%)	7 (35%)
**Fraction genome altered**	0.08 (0.03, 0.18)	0.06 (0.02, 0.15)	0.08 (0.03, 0.15)	0.10 (0.04, 0.21)	0.22 (0.08, 0.45)
**TMPRSS2:ERG status**					
ERG-pos	140 (48%)	47 (45%)	68 (52%)	19 (54%)	6 (30%)
ERG-neg	152 (52%)	58 (55%)	64 (48%)	16 (46%)	14 (70%)
**PTEN copy number status**					
diploid	206 (71%)	77 (73%)	98 (74%)	19 (54%)	12 (60%)
hetloss	39 (13%)	10 (10%)	16 (12%)	7 (20%)	6 (30%)
homdel	47 (16%)	18 (17%)	18 (14%)	9 (26%)	2 (10%)

Supplementary Table 2.

a.

AZIN1 Localization	Nuclear	Cytoplasmic
Benign tissue (n = 26)	0 (0%)	10 (39%)
Prostate Cancer (n = 202)	98 (49%)	104 (51%)
		P < 0.001

b.

Characteristic	Overall	By AZIN1 expression	
		Cytoplasmatic	Nuclear
**N**	202	111	91
**Age (years)**	62.0 (57.0, 67.0)	63.0 (58.0, 67.0)	61.0 (57.0, 66.0)
**Gleason grade**			
<7	92 (46%)	56 (50%)	36 (40%)
3+4	69 (34%)	37 (33%)	32 (35%)
4+3	14 (7%)	6 (5%)	8 (9%)
8	10 (5%)	6 (5%)	4 (4%)
9-10	17 (8%)	6 (5%)	11 (12%)
**Stage, pT**			
pT2	148 (73%)	83 (75%)	65 (71%)
pT3	54 (27%)	28 (25%)	26 (29%)
**Stage, pN**			
N0	202 (100%)	111 (100%)	91 (100%)
**Cribriform morphology**	32 (16%)	19 (17%)	13 (14%)

c.

AZIN1 Expression	Low Expression	High Expression
Benign tissue (n = 26)	16 (62%)	10 (38%)
Prostate Cancer (n = 202)	111 (55%)	91 (45%)
		P = 0.68

Supplementary Table 3.

a. AZIN Mutant Primer Sequences

AZIN Mutation	Direction	Primer Sequence 5'-->3'
S367G	Forward	GATCAAATTGTGGAAGGCTGTCTTCTTCCTG
	Reverse	CAGGAAGAAGACAGCCTTCCACAATTTGATC
S367A	Forward	CTTGATCAAATTGTGGAAGCCTGTCTTCTTCCTGAGC
	Reverse	GCTCAGGAAGAAGACAGGCTTCCACAATTTGATCAAG
S367N	Forward	GATCAAATTGTGGAAAACTGTCTTCTTCCTGAGC
	Reverse	GCTCAGGAAGAAGACAGTTTTCCACAATTTGATC

b. N-Terminal Fluorescent Protein Primer Sequences

N-Terminal Fluorescent Fusion Protein	Direction	Primer Sequence 5'-->3'
6x His-Clover-Thr-AZIN1	Forward	CCAAGCTTCGAATTCTTAT TAAGCTTCAGCGGAAAA
	Reverse	TGGACGAGCTGTACAAG CTGGTTCCGCGTGGTAGT AAAGGATTTATTGATGATGC
6x His-mRuby2-HRV3CP-AZ	Forward	GCCAAGCTTCGAATTCTTACTACTCCTCCTCCTCTCC
	Reverse	ATGGACGAGCTGTACAAGCTTGAAGTCCTCTTTCAGG GACCCgtgaaatcctccctgcagc

c-Terminal Fluorescent Protein Primer Sequences

C-Terminal Fluorescent Fusion Protein	Direction	Primer Sequence 5'-->3'
6x His-AZIN-Thr-Clover	Forward	ctgtacttccagggcATGAAAGGATTTATTGATGATGCAAAC
	Reverse	Ccttgctcaccatgcgactaccacgcggaaccagctt AGCTTCAGCGGAAAAGCTG
6x His-AZ-HRV3CP-mRuby2	Forward	ctgtacttccagggcGTGAAATCCTCCCTGCAG
	Reverse	cccttagacaccatgccgggtccctgaaagaggacttcaagc TCCTCCTCCTCTCCCGAA

Supplementary Table 4.

sgRNA 1	Target sequence	CACCGCTCGGCGATCTGGTCGCTG
	Reverse complementary	AAACCAGCGACCAGATCGCCGAGC
sgRNA 3	Target sequence	CACCGCGCGCTACTCACGAAGCGGG
	Reverse complementary	AAACCCCGCTTCGTGAGTAGCGCGC
sgRNA 4	Target sequence	CACCGCGGGGCCTTCAAGACGCGGA
	Reverse complementary	AAACTCCGCGTCTTGAAGGCCCCGC
sgRNA 6	Target sequence	CACCGCGCCGCGCTACTCACGAAGC
	Reverse complementary	AAACGCTTCGTGAGTAGCGCGGCGC
sgRNA 7	Target sequence	CACCGACGCCACGTACGCCAGATAC
	Reverse complementary	AAACGTATCTGGCGTACGTGGCGTC
sgRNA SPL (targeting a myosin 9 splicing factor)	Target sequence	CACCGTAGATGCCTTGTCGGCCTGCGG
	Reverse complementary	AAACCCGCAGGCCGACAAGGCATCTAC

SUPPLIMENTARY FIGURE LEGENDS

Supplementary Fig. 1. Uneditable AZIN1 does not alter gene expression. HEK293 cells were transfected with plasmids expressing fluorescent protein (Empty), wt-AZIN1, ed-AZIN1, or uned-AZIN1 for 24h and the RNA were extracted and analyzed by RNA sequencing by principal component analysis.

Supplementary Fig. 2. Endogenous AZIN is localized at the cytoplasm. PC3 (**a**) and HEK293 (**b**) cells were stained for AZIN1 and analyzed by confocal microscopy.

Supplementary Table 1. Characteristics of men with primary prostate cancer in The Cancer Genome Atlas, by edAZIN1. Cells show count (percent) or median (interquartile range).

Supplementary Table 2. (**a-c**) Summary of the findings from tissue microarrays from 202 prostate cancer patients and 26 adjacent benign matched tissue controls analyzed by IHC by staining for AZIN1 localization and counter staining with hematoxylin to measure the expression level of AZIN1.

Supplementary Table 3. (a-c) Gene, Primers and Vector sequences for constructing bacterial expression vectors of AZIN1 mutants.

Supplementary Table 4. Sequences for Myosin-9 targeting guide RNAs.
